# Periscope of Medical and General Science in Their Relations to Dentistry

**Published:** 1866-02

**Authors:** Geo. J. Ziegler


### PERISCOPE OF MEDICAL AND GENERAL SCIENCE IN THEIR RELATIONS TO DENTISTRY.

BY GEO. J. ZIEGLER, M.D.

*Reflex Action.* By C. HANDFIELD JONES, M.B., F.R.C.P., F.R.S., Physician to St. Mary's Hospital, and Lecturer on Medicine.—"The chief question with regard to reflex or inhibitory paralysis is, does it depend on an influence exerted on the tissue of the nervous centres, or on excitation of the vaso-motor nerves of their bloodvessels, by which the latter are thrown into a state of anæmiating spasm? The latter is the view maintained by Brown-Séquard. It has in its favor the possibility of explaining on known grounds the occurrence of the paralysis; but against it there are considerations which seem to me greatly to preponderate. It is difficult to conceive that a spasm of remote origin should be limited to such a very small extent of vessels as would be contracted in some instances—*e.g.*, palsy of one-sixth nerve, ptosis of one eyelid. Spasms produced by reflex excitation tend mostly to assume a diffuse character. It is almost impossible to believe that a spasm of vessels should be so persistent as the hypothesis requires. In some instances, it would be necessary to suppose that the anæmiating spasm had continued for weeks or months. Moreover, we know from the results of embolism that any arterial obstruction continued above a few days in the brain is followed by degeneration and decay of the tissue. This would certainly have occurred in some of the cases which were of long standing, if the palsy had depended on anæmia of the nervous tissue. Now, even supposing the excitation to remain in operation all the time, there is no doubt that the muscular contractility would, sooner or later, become exhausted, and the contraction therefore gives way to dilatation. Bernard says expressly that reflex actions of the vaso-motor nerves are manifested by contraction of the vessels, followed by dilatation. Besides, on Brown-Séquard's view, the cessation of the irritation ought always to be followed speedily by cessation of the paralysis, the vessels again admitting blood into the previously anæmic part; whereas, we know that, in many cases, the paralysis continues long, or even does not come on at all, until after the irritation has ceased to act. Not only so, but a curative effect is sometimes produced by a stimulation of a different kind, as the interrupted current, which would tend to contract vessels. Again, if it be admitted that a nervous centre may be functionally paralysed from such causes as catarrh, overexertion, insolation, etc., a considerable argument is supplied for the view that a similar state may be induced also in a reflex manner. If, as I shall presently show, neuralgic pain is to be regarded as a condition akin to paralysis, we have, in the very frequent instances of reflected pains we meet with, examples of morbid action closely allied to inhibitory. In these, however, it can hardly be thought that the nervous centre affected by the primary irritation is necessarily anæmic. Moreover, it may be remarked that the same cause which gives rise to inhibitory paralysis may also produce very different effects, as tetanic spasm, which certainly does not depend on an anæmic state of the centre.

"Assuming, then, that in reflex or inhibitory paralysis, both in its more and less typical forms, the essential change is in the cells of the nervous tissue itself, and not in the vessels, we will proceed to consider some other instances of this kind of disorder. Dr. Watson refers to the production of amaurosis without visible change in the eye, in consequence, apparently, of irritation of the dental nerves, the blindness ceasing after the extraction of some teeth which had grown irregularly. He quotes from Mr. Laurence an interesting case, in which the extraction of a carious tooth, with a splinter of wood projecting from one of its fangs, procured the restoration of the sight of the eye on the same side, which had been entirely lost for thirteen months. Improvement in vision commenced immediately, and recovery was perfect by the ninth day. It is impossible that the complete amaurosis in this case could have been due to retinitis, as this would have certainly taken a much longer time to subside, and would, assuredly, after lasting so long, have left behind irreparable damage. It is worth remarking for future reference as to the nature of neuralgia, that the amaurosis was attended with severe pain in the same side of the head and face. This I interpret as another paralytic phenomenon. Deafness is certainly a rarer result of inhibitory irritation than amaurosis, but the following case appears to be an example of it. It is related by M. Vautier, '*Ann. par Jamain*,' 1861, p. 90:—A lady, aged 54, spare and of a nervous temperament, was attacked, about four months before he saw her, with pain radiating into almost all the teeth, as well as into the muscles of the anterior and left side of the head. The eye of this side was in an almost constant state of lachrymation, and she had become completely deaf of this ear. She was sleepless and without appetite. After the extraction of the left wisdom tooth, which was a little loose and painful, the deafness immediately ceased, and the neuralgia disappeared. The rapidity of the change proves, I think, that the deafness was not the result of inflammation or any structural change in any part of the organ, but a nerve-disorder homologous to the existing neuralgia. It appears to me somewhat remarkable that, while reflected pains are so abundantly common, anæsthesia or numbness of like origin is decidedly rare. Both in the more acute and chronic forms of paraplegia produced by cold and wet, Graves states that there is much less impairment of sensation than of motion, and the loss of sensation is never so complete as in paraplegia from disease of the spine. The much greater tendency to impairment of motor than of sensory power in almost all kinds of nervous diseases, organic as well as inorganic, is a remarkable and unexplained fact. Schuh observes that motor nerves require for the exercise of their function a much greater perfection of structure than sensory do, and the restoration of the integrity of the nerve is less easily affected.

"Morbid impressions conveyed from the internal organs are well known to produce depressing effects on the nervous system, just as *per contra* impressions of an opposite kind are attended with a sense of satisfaction and 'wohlsein.' It is known to physiologists that the cravings of an empty stomach are much better appeased when some solid, though innutritious material, as earth or sawdust, is mixed with the liquid oil or honey on which some savage tribes are at times reduced to feed, than when the latter are taken alone. On the same ground, the Canadian 'voyageurs' tighten their famine girdles to the last holes when the vacuum so abhorrent to nature generally is felt and there is nothing to fill it. In a case under my own care, sensory paralysis was the result of gastric irritation. A female, aged 32, had been ailing a long while. She suffered with stomach disorder of sub-inflammatory character, marked by anorexia, pain after food, and vomiting directly after eating. There was also severe headache, extending from the forehead to the back of the head, constant and not affected by position. Attacks occurred two or three times a week, or oftener, in which the left side of the face, and tongue, and left arm became numb, the numbness beginning in the tips of the fingers, and extending upwards to the face, lips, and tongue. At these times, her speech became thick, and continued so for an hour. Under treatment, directed to relieve the inflammatory condition of the stomach, the reflected disorder diminished considerably. The numbness of the thigh, which occurs in some cases during the descent of a calculus from the kidney to the bladder, is another instance of inhibitory anæsthesia.

"More frequent than disorders of the sensory nerves, are those where the intellectual centres are affected. Mr. Langston Parker says, 'In some persons, where the powers of the mind are naturally acute, they are singularly depressed after a full meal; some lose the faculty of thinking at all, others become deficient in judgment, and in others, again, memory is quite lost.' I was called to the case of a gentleman, in the course of last year, who was suffering from a sub-acute inflammatory affection of the stomach, who completely lost the faculty of memory after taking a teacupful of food, yet this patient was remarkable for his mechanical genius. A less portion of food did not impair his mind, which invariably became more powerful and clear from leeching or fomenting the epigastrium. Mr. Grantham relates a case (*Brit. Med. Jour.* June 7, 1862,) in which a man, aged 48, who died with symptoms apparently of cancer of the stomach, had been subject to paroxysms of violence, both mental and bodily, occurring more or less after dinner and supper. These exacerbations had gradually increased in severity from the commencement. The following case has been kindly communicated to me by Dr. Palmer:—Mrs. H., aged 50, has had a large family; still menstruates occasionally. Is temperate and regular in her habits, taking only two or three glasses of ale daily. Is subject to tremor of hands and tongue when fatigued or excited at any time; is rather dyspeptic. October 28th, complained of pain of back, and sleeplessness, and general debility, but appeared perfectly rational, saying that the cause of her not sleeping was that some actors had been sitting up late in the next house, singing and making a noise. I really thought it was true, until her children assured me it was a delusion. As usual, when anything ails her, she is in a tremor all over; tongue clean, pulse weak, but not hurried, skin perspiring, bowels well open. Complains of pain and fulness of right side in region of liver, which she says feels swollen; the extent of dulness has increased. Is quite cheerful and happy. No cause for this attack discoverable. Ordered full stimulating diet and tr. opii min. xv 3tis hôris. Next day, pain of back was gone; other symptoms were worse; she was weaker and sick; saw cats, dogs, fires, etc. Still answers questions, apart from the delusions quite rationally. Still smiling and merry. Not an hour's sleep last night. It was determined to try the plan fully, so the doses were doubled. Next day, gets worse and worse; has more delusion, but is as happy as ever. Next day, condition same; rather more pain in right side. The plan was now changed, her meat and stimulus were discontinued, and three grains of calomel were given. In three or four hours after taking the pill the delusions began to abate; three or four hours later, the bowels acted freely (they had been confined for three days before); very soon after she fell asleep, and slept for six hours continuously, having had, practically, no sleep for five nights. On awaking, the delusions were all but gone, and she speedily got quite well. In this instance, the depressing and disordering influence of the loaded state of the liver and bowels on the hemispherical ganglia is plainly apparent. The disorder was of the nature of a paresis, but cannot be attributed to anæmia of the centres. Dr. Tilbury Fox records a case in which symptoms identical with those of delirium tremens were brought on by exposure to cold and wet. The patient was a perfectly temperate man, aged 45, but he seems to have had an hereditary predisposition to cerebral disorder. Here, also, the nervous disorder was evidently of paralytic character, the functional power of the brain was evidently impaired, and the same is true of the following instance: A lady, who had been a very excellent and affectionate mother, displayed unequivocal symptoms of moral insanity. She continued in this state some time, and was at length restored by having a great number of stumps of decayed teeth extracted, which, however, did not seem to have caused any marked pain. In his excellent paper on symptomatology of worms, Dr. Heslop describes headache and giddiness as prominent symptoms. The headache is almost constant, and sufficiently severe, without being excruciating, to render life, if not a burden, at least unhappy. The giddiness is so severe that the patient often staggers about like one intoxicated, and when this symptom is present to a less degree, there is still almost continuously a sense of confusion and insecurity, which renders walking a serious effort. Both these symptoms indicate, plainly, a state of depression, of partial paresis, of some of the encephalic centres. The influence exerted by the uterus on the nervous centres, even on those of the highest order, is very considerable, and, from the great variety of effects produced, demands specially to be noticed here. In some cases, the gravid condition of the organ evidently causes derangement and depression of one or more parts of the nervous system. Thus, it may produce severe frontal neuralgia, gastric hyperæsthesia of the most intense kind, chorea, hemiplegia, paraplegia, deafness, amaurosis, or a state of complete cerebral torpor in which the patient appears like a living automaton, and all indications of mind are in abeyance, or insanity. Instances of all these various forms of disorder are given by Dr. Lever, in his paper in 'Guy's Hospital Reports,' vol. v., 1847. The histories of several of the cases seem to make it highly improbable that the manifold symptoms could depend on anæmia of the nervous centres produced by arterial spasm. This conclusion is strongly supported by the fact that the same condition sometimes acts as a beneficial stimulus, improving a naturally irritable temper, and invigorating general or particular nutritive actions. Dr. Churchill states that pregnancy occasionally relieves mental derangement, and mentions having seen a lady who was in a state of confirmed melancholia, which disappeared entirely on her becoming pregnant. Asthma sometimes disappears during pregnancy, and digestion is occasionally more vigorous then than in the unimpregnated condition. I am acquainted with one remarkable case in which albuminaria, which had existed for at least fifteen months before pregnancy, ceased during its continuance, at the same time that the health improved and the body gained flesh, which had been observed in former pregnancies. Here, there can be no question that a beneficial influence was exerted on the renal plexus—an event which is more remarkable as the exact converse is the more ordinary occurrence."—*Med. Times & Gazette, and Dental Cosmos.*

